# Improving polygenic risk prediction from summary statistics by an empirical Bayes approach

**DOI:** 10.1038/srep41262

**Published:** 2017-02-01

**Authors:** Hon-Cheong So, Pak C. Sham

**Affiliations:** 1 School of Biomedical Sciences, Chinese University of Hong Kong, Shatin, Hong Kong; 2 KIZ-CUHK Joint Laboratory of Bioresources and Molecular Research of Common Diseases, Kunming Institute of Zoology and Chinese University of Hong Kong, China; 3 Department of Psychiatry, University of Hong Kong, PokFuLam, Hong Kong; 4 Centre for Genomic Sciences, University of Hong Kong, PokFuLam, Hong Kong; 5 State Key Laboratory for Cognitive and Brain Sciences, University of Hong Kong, PokFuLam, Hong Kong; 6 Centre for Reproduction, Development and Growth, University of Hong Kong, PokFuLam, Hong Kong

## Abstract

Polygenic risk scores (PRS) from genome-wide association studies (GWAS) are increasingly used to predict disease risks. However some included variants could be false positives and the raw estimates of effect sizes from them may be subject to selection bias. In addition, the standard PRS approach requires testing over a range of *p*-value thresholds, which are often chosen arbitrarily. The prediction error estimated from the optimized threshold may also be subject to an optimistic bias. To improve genomic risk prediction, we proposed new empirical Bayes approaches to recover the underlying effect sizes and used them as weights to construct PRS. We applied the new PRS to twelve cardio-metabolic traits in the Northern Finland Birth Cohort and demonstrated improvements in predictive power (in *R*^2^) when compared to standard PRS at the best *p*-value threshold. Importantly, for eleven out of the twelve traits studied, the predictive performance from the *entire* set of genome-wide markers outperformed the best *R*^2^ from standard PRS at optimal *p*-value thresholds. Our proposed methodology essentially enables an automatic PRS weighting scheme without the need of choosing tuning parameters. The new method also performed satisfactorily in simulations. It is computationally simple and does not require assumptions on the effect size distributions.

Traditionally, for complex diseases with substantial heritability, the prediction of disease risk has been largely based on family history and clinical risk factors. However, with the advent in genotyping technologies, an increasing number of susceptibility variants for complex diseases have been identified. In particular, genome-wide association studies (GWAS), which allow interrogation of more than a million single nucleotide polymorphisms (SNPs) in the genome, has become a very popular and useful tool in deciphering the genetic basis of complex diseases^1^,^2^. Besides revealing the underlying pathophysiology of complex diseases, susceptibility variants may also be useful in improving the prediction of disease risks or trait values.

Polygenic risk scores (PRS) are usually constructed as a weighted sum of allele counts, the weights being given by log odds ratios or linear regression coefficients from univariate regression tests. A simpler approach by weighting each marker by +1 or −1 is also possible^3^. It is natural to question if we could improve the weighting method to enhance the predictive power. Genomic prediction of disease risks might have implications in designing more individualized preventive or screening strategies for patients, for example earlier screening for breast cancer may be warranted for those having a high genetic risk for the disease^4^. As individual-level genomic data is often not available to researchers due to privacy concerns, we focus on ways to improve risk prediction using GWAS summary statistics, which are freely accessible for a wide range of traits. In practice, even when original genotype data can be accessed, it is often not possible to gather a sample size comparable to that of modern GWAS meta-analyses.

Traditionally only the top established variants are considered in risk prediction. However, variants with smaller effects may also aid prediction. Purcell *et al*.^5^ proposed a polygenic risk score (PRS) approach to dissect the genetic architecture of schizophrenia and bipolar disorder by including a large number of genetic markers for prediction. The PRS approach has become popular in recent years and has been widely applied to different traits^6^,^7^,^8^.

To our knowledge, only two previous studies focused on how to improve genomic risk prediction from summary statistics. A Bayesian genomic risk prediction method known as LDpred^9^ was recently proposed for this purpose by taking into account linkage disequilibrium (LD) among markers. The method uses a reference LD panel and was shown to improve predictive power for selected traits. In a very recent piece of work, Mak *et al*. proposed a new way of constructing PRS by weighing the estimated effect sizes with the local true discovery rate^10^. All markers can then be included in the PRS. It was reported that the method achieves comparable performance to the standard PRS using the best *p*-value threshold.

In this study we proposed a different PRS weighting scheme compared to LDpred^9^ or Mak *et al*.^10^, using empirical Bayes estimation of the underlying effect sizes. Compared to LDpred, our method is computationally simpler as no Monte Carlo Markov Chain (MCMC) procedures are involved. Unlike LDpred, the newly proposed method does *not* require any distributional assumptions of effect sizes. Moreover, both the standard PRS and LDpred necessitate a tuning parameter to be chosen, and require testing over a range of *p*-value thresholds or fractions of causal variants. The choice of such thresholds is usually arbitrary, and the predictive performance estimated from the optimized threshold may be subject to an optimistic bias. It is therefore desirable to develop a new PRS weighting method such that all markers can potentially be included in the prediction model, obviating the choice of any thresholds. As we will describe later, for 11 out of 12 actual disease traits studied, the predictive performances of our new approach with the entire set of genome-wide markers outperformed the best *R*^2^ from standard PRS at the optimal *p*-value thresholds, which are not known in advance. We also showed that our approach outperformed Mak *et al*.’s method in actual GWAS datasets.

## Materials and Methods

### Standard polygenic risk estimates

Polygenic risk scores are usually constructed from a weighted sum of allelic count allelic counts. Without loss of generality, we assume the allelic counts have been standardized, and the PRS can be given by 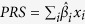, where 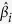 is the log odds ratio or the estimated regression coefficient from a linear or logistic regression.

### LD-clumping followed by p-value thresholding

In practice, due to the presence of linkage disequilibrium (LD) between genetic markers, markers are usually LD-pruned before construction of PRS. It is common to include variable selection in the pruning procedure, such that the more significant makers are preferentially retained. This kind of algorithm is implemented in PLINK, also known as “LD-clumping”. In this study, for standard and the three other PRS weighting methods, we first applied LD-clumping with an *r*^2^ threshold of 0.25 to all SNPs, followed by *p*-value thresholding in the test set.

### Estimation of the true effect sizes by Tweedie’s formula and its variants

Assume that a GWAS or meta-analysis of GWAS was done and we tested the association of each variant with the phenotype by a regression analysis. We formulated the problem as recovering the “*true*” *z-*statistic (i.e. the *z*-statistic one would obtain if there were no random noise; reflecting the true effect size) from a set of *observed z*-statistics. The *z*-statistics can then be converted to variance in liability (or heritability) explained as described in So *et al*.^11^, which can then be converted to corrected estimates of 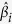.

It is useful to consider the following model for our problem. The observed *z*-statistics are denoted by *z*. We assume that


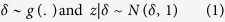


where *δ* is the underlying true effect size. *δ* = 0 for null variants and is non-zero for the truly associated variants.

Efron^12^ proposed an empirical Bayes approach to recover *δ*:


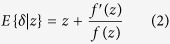


where *f(x*) is the is probability density function of *x.* The same formula was discovered by Tweedie in 1950 s, hence also named the Tweedie’s formula^13^. We employed a kernel density function to estimate *f(x*) as described in So *et al*.^11^. The kernel density estimate with kernel *K* is given by


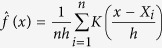


where *h* is the bandwidth or smoothing parameter. The computation was performed using the R function “density”, with the default settings using a Gaussian kernel. An important advantage of the Tweedie’s formula is that it only involves estimation of the marginal density *f(x*), hence avoiding the need to derive the distributions of z-statistics under H_0_ and H_1_. This approach does *not* require any particular choice of the prior distribution of *δ*.

Using the Tweedie’s formula, the corrected estimates of *β*_*i*_ (denoted as 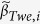) can be expressed as


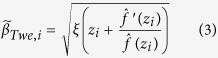


where *ξ* is a function to convert the z-statistics to variance in liability explained (V*g*)^11^,^14^.

The Tweedie’s formula shrinks effect size estimates towards zero, but unlike some other procedures such as LASSO^15^, the Tweedie’s method does not perform variable selection by forcing effect size estimates to zero. In the GWAS setting, in general we expect only some but not all markers to be associated with the outcome. We propose another estimator of the effect size as follows:


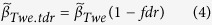


where *fdr* is the local false discovery rate^16^, the probability of null given the observed *z*-statistic. (1-*fdr*) is hence the local true discovery rate. This method weighs each effect size estimate from Tweedie’s formula by the probability of being non-null. In practice, it will further shrink effect sizes towards zero and a portion of the effect sizes will become zero, as the local fdr equal one for some markers.

We also include another estimator that was recently developed by Mak *et al*.^10^


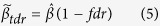


in which the regression coefficients are weighted by the local true discovery rates. For the estimation of fdr, we employed *locfdr*, an R package based on the methodology described by Efron^16^. We observed that sometimes the *locfdr* algorithm failed to converge in the presence of more extreme *z*-statistics. We hence assumed a local fdr of zero for genetic variants with 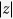 >= 6 (corresponding to a *p*-value of 1.97 × 10^−9^). For simplicity, we will also use 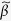 in the following text to denote the corrected effect size estimates in general.

### Polygenic risk score constructed from corrected effect size estimates

Following the previous derivations, we wish to test if PRS directly constructed from the corrected effect size estimates improve prediction. The new PRS can be expressed as 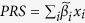, where 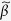 can be obtained from formula (3), (4) or (5).

### Polygenic risk prediction with LDpred

LDpred is a recently developed algorithm which takes into account the LD among genetic markers for polygenic risk prediction. The effects of markers are assumed to follow a Gaussian distribution. In an infinitesimal model, all markers are assumed to be causal and the marker effects follow the distribution 
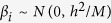
, where *M* is the total number of markers and *h*^2^ is the total heritability explained by the panel markers. The algorithm also allows for a non-infinitesimal model, in which only a fraction *p* of all makers are causal. A Gaussian mixture prior is assumed in this case in which 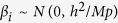 with probability *p* and 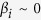 with probability (1-*p*).

LDpred computes the posterior mean effects of markers, taking into account the LD structure. The effect sizes can be computed for different proportion of causal markers *p*. An approximate MCMC Gibbs sampler is used to estimate the posterior mean for the non-infinitesimal case while an analytic solution is available for the infinitesimal case. In this study, we followed all the recommended parameters set by the authors of LDpred. The LDpred program was downloaded from https://github.com/bvilhjal/ldpred.

### Simulations with independent SNPs

We first performed simulations with independent SNPs. A liability threshold model was assumed in the simulations. A panel of 20,000 independent SNPs was simulated, and 2.5% of the SNPs were set to be causal. The total heritabilities explained were set at 0.15, 0.35 or 0.55. The training sample sizes were set at 5000, 10000, 15000 or 20000 respectively. The test sets were of equal size to the train sets. We assumed a minor allele frequency (MAF) of at least 0.05 and uniform MAF. The true regression coefficients were assumed to follow a normal distribution with mean zero and variance equal to the average heritability per marker, i.e. 
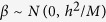
, where *M* is the total number of causal markers. Liability (or equivalently the quantitative phenotype) was simulated as 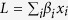 where *x* denotes the standardized genotype counts. To reduce memory and computational costs, the prevalence was set at 0.45 with people exceeding the liability threshold assuming to be affected. An equal number of cases and controls were then sampled.

PRS were computed from the training set, using the uncorrected and each of the corrected coefficient estimates (3), (4) and (5). Predictive performances were assessed in the testing set in terms of prediction R-squared and area under the receiver operating characteristic curve (AUC). Simulations were repeated for 20 times.

### Simulations using real genotype data

Next we performed larger scale simulations using real genotype data. Raw genotype data was obtained from a GWAS of cardio-metabolic traits from the Northern Finland Birth Cohort (NFBC) 1966^17^. The data was accessed from dbGaP (accession number phs000276.v2.p1). Details of the study design are described elsewhere^17^. Briefly, the study subjects were from Northern Finland and all traits in this study were measured at 31 years of age. Genotyping was done by the Infinium 370cnvDuo array. Standard quality control procedures were performed. Briefly, genetic variants with missing rate >10%, minor allele frequency <0.01 and with significant deviation from Hardy-Weinberg equilibrium (*p* < 1e-5) were excluded. Individuals with genotyping rates <90% are excluded from analyses. In each training set, linear regression was carried out with the top 10 principal components as covariates. After quality control procedures, 334458 variants and 5402 individuals were retained for further analyses.

We simulated phenotypes under three gross categories of genetic architecture:

1. An infinitesimal model. All markers were assumed to be causal. The effect sizes were simulated by 
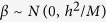
, and *h*^2^ was set at 0.1, 0.2, 0.3 or 0.4. This model matches exactly with the prior assumed in LDpred when all markers are causal.
2. A model with only a limited number of large-effect variants. We simulated 5, 10, 20, and 25 large-effect markers each with heritability explained = 0.6%. All other markers were null.
3. A model in which both small and larger effects were present. Two sets of casual varaints were simulated and then combined as described below.

The first set of causal variants were simulated under a double exponential (*i.e.* Laplace) model by 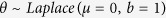, where *μ* and *b* are the location and scale parameters respectively. Different fractions of causal markers (2.5%, 1%, 0.25% or 0.1%) were assumed. The effects *θ* were further scaled by 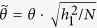, where 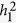 denotes the total variance explained by the first set of casual variants and *N* denotes the corresponding number of variants. This is to ensure that the total variance explained summed up to the desired level of heritability.

The second set of simulated causal variants had larger effect sizes on average. Their variance explained was assumed to follow a uniform distribution in the interval [0.4%, 0.8%]. The total heritability explained by the second set of variants was 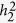 and 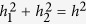. The total heritability *h*^2^ was set at 0.1, 0.2, 0.3 or 0.4. Supplementary Table 3 shows the details for the 16 scenarios simulated under category (3).

Prediction was performed using the standard PRS, three other corrected effect size estimates [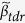, 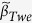 and 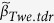] and LDpred. LDpred does not require pruning of SNPs. We followed all the default parameters when running LDpred. Briefly, the LD radius (the number of SNPs on either side of the focal SNP for which LD is adjusted) was set to *M/*3000, and the following fractions of causal markers were considered as preset by the program: 1, 0.3, 0.1, 0.03, 0.01, 0.003, 0.001, 0.0003 and 0.0001. For each simulation, the testing set (obtained from cross-validation, as described below) was used as the reference LD panel.

For methods other than LDpred, we performed LD-clumping using the following PLINK command: –clump-p1 1 –clump-p2 1 –clump-r2 0.25 –clump-kb 250. Note that the algorithm preferentially selects the most significant markers. On average around 110,000 variants were retained after clumping. We set *p*-value thresholds at 1e-5, 1e-4, 5e-4, 1e-3, 5e-3, 1e-2, 0.03, 0.05, 0.1, 0.2, 0.3, 0.4, 0.5, 0.6, 0.7, 0.8, 0.9 and 1.

We employed 5-fold cross-validation (CV) to estimate the predictive performance of polygenic scores, using the uncorrected and corrected effect size estimates. Summary statistics were derived from the train sets. Cross-validation was repeated 4 times, producing a total of 20 pairs of train sets and test sets.

### Application to twelve cardio-metabolic traits in the Northern Finland Birth Cohort

We also applied the five different PRS weighting methods to twelve anthropometric and metabolic traits in the NFBC. The traits included triglycerides (TG), high-density lipoprotein (HDL), low-density lipoprotein (LDL), insulin (INS), glucose (FG), C-reactive protein (CRP), body mass index (BMI), waist-hip ratio (WHR), systolic blood pressure (SBP), diastolic blood pressure (DBP) and body height and weight. Five-fold CV (repeated 4 times) was used to assess predictive performance of different polygenic scoring methods.

Quality control and basic GWAS association analyses were performed using PLINK 1.9^18^. Other analyses were performed in R3.2.2. R code will be available on the first author’s website (https://sites.google.com/site/honcheongso/).

## Results

### Simulations with independent SNPs

The results of simulations with independent SNPs are shown in Tables 1, 2 and Supplementary Tables 1, 2. We tested the predictive performance of PRS constructed by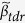, 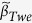 and 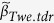 in simulations. Generally all three estimators performed similarly, with 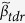 having a slight advantage over the other estimators. Compared to the standard PRS at the optimal *p*-value threshold, 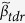 slightly outperform standard PRS for linear traits, and for binary traits all three estimators showed mild improvements in AUC compared to the standard method.

When all the SNPs were included in the PRS, all three estimators performed very well and they outperformed the standard PRS method by a large margin. In addition, their performances were highly comparable to and sometimes outperformed the standard PRS at the optimal thresholds (Table 2 and Supplementary Table 2).

### Simulations using real genotype data

Table 3 and Supplementary Tables 4, 5 show the simulation results with real genotype data from the NFBC. Under an infinitesimal model in which all variants are casual with normally distributed effects, LDpred performed very well as expected (Supplementary Table 4). LDpred outperformed other methods except for *h*^2^ = 0.1. Interestingly, 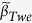 achieved the best predictive performance in this case. The standard PRS was the second best in general.

Under another model in which only few large-effect variants were present, the predictive performances of the five methods considered were much closer (Supplementary Table 5). When considering the performances at the optimal threshold of *p* (either *p*-value or fraction of causal variants), LDpred slightly outperformed the other methods when the number of causal variants (*N*_causual_) was 5, 15 or 20. Nevertheless, other methods especially 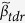 followed very closely. 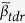 was the best method when *N*_causual_ was 10. However, when we consider prediction from *all* markers, 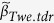 was the clear winner for all *N*_causual_.

The third type of genetic architecture involves a mixture of variants with small and larger effects, and is probably a more realistic model. If we consider the predictive performance at the optimal *p* thresholds, there is no clear winner across all scenarios. However, compared to the standard PRS, methods using corrected effect sizes (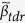, 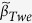, 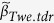 and LDpred) generally performed better (Table 3). When the total heritability was low at *h*^2^ = 0.1, 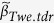 and 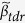 performed well while LDpred tended to have lower prediction *R*^2^ than other approaches. LDpred however demonstrated better performance when the proportion of causal variants = 2.5%. Overall the results were mixed and 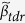, 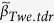 and LDpred were the best performing estimators in different scenarios. On the other hand, when *all* markers were included (i.e. no choice of *p* threshold), 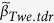 unanimously outperformed other methods and the predictive performances were very close to or identical to the prediction *R*^2^ at the optimal *p* threshold.

### Application to twelve cardio-metabolic traits in the Northern Finland Birth Cohort

**Figure 1:**
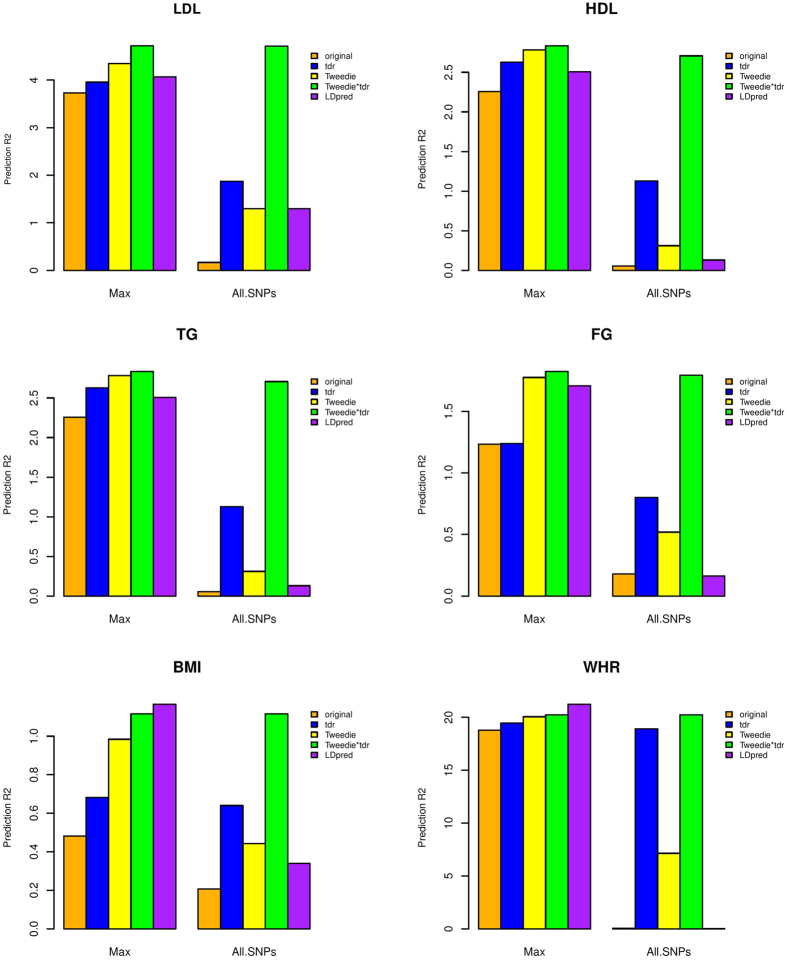
Predictive performance of standard PRS and four other PRS weighting schemes on lipids, fasting glucose, body-mass index and waist-hip ratio in the Northern Finland Birth Cohort (Orange: standard PRS; blue: weighting by 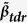; yellow, weighting by 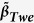; green, weighting by 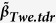; purple, weighting by LDpred). For all methods except LDpred, we first applied LD-clumping with an *r*^2^ threshold of 0.25 to all SNPs. “Max” refers to the maximum prediction *R*^2^ achieved after optimizing over a range of *p*-value thresholds or fractions of causal variants. “All.SNPs” refers to the predictive performance using all SNPs after LD-clumping, except for LDpred where no clumping was performed. All predictive performances were measured by *R*^2^ in %.

**Figure 2:**
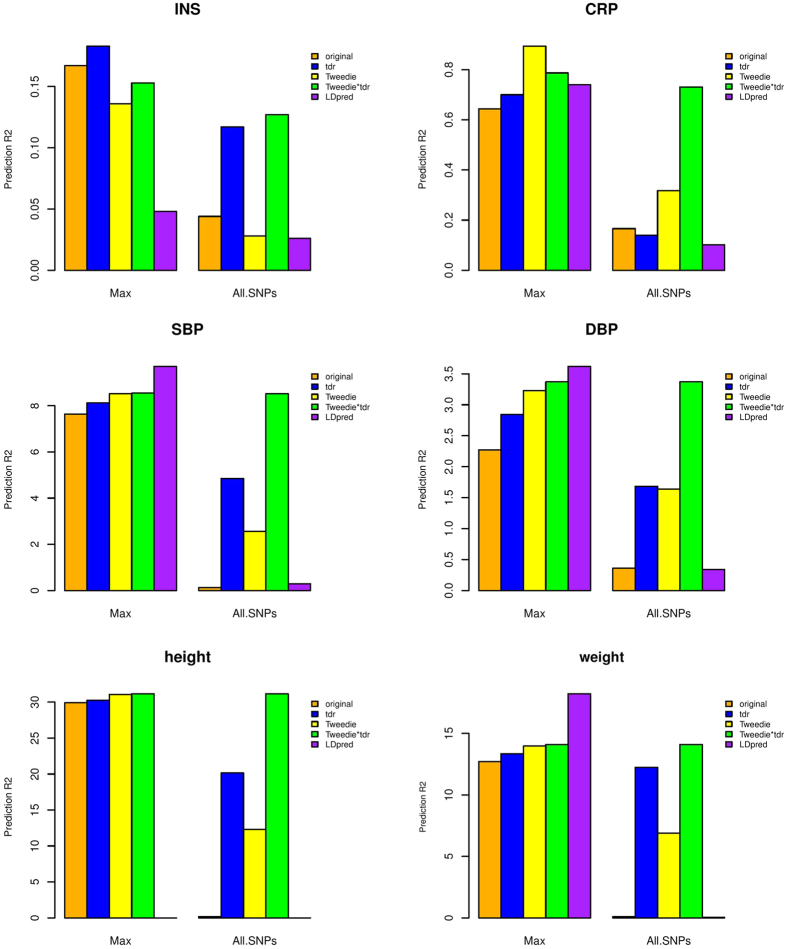
Predictive performance of standard PRS and four other PRS weighting schemes on fasting insulin, C-reactive protein, systolic and diastolic blood pressure, height and weight in the Northern Finland Birth Cohort (Orange: standard PRS; blue: weighting by 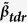; yellow, weighting by 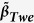; green, weighting by 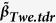; purple, weighting by LDpred). For all methods except LDpred, we first applied LD-clumping with an *r*^2^ threshold of 0.25 to all SNPs. “Max” refers to the maximum prediction *R*^2^ achieved after optimizing over a range of *p*-value thresholds or fractions of causal variants. “All.SNPs” refers to the predictive performance using all SNPs after LD-clumping, except for LDpred where no clumping was performed. All predictive performances were measured by *R*^2^ in %.

The average prediction *R*^2^ from 5 × 4 CV are shown in Table 4 and Figs 1, 2. Methods using corrected effect sizes in most cases outperformed the original PRS approach, except in the prediction of INS. The relative improvements for some traits were quite considerable. For example, the maximum prediction *R*^2^ improved from 1.23% to 1.82% (using 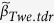) for FG, from 0.48% to 1.12% (using 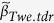) and 1.17% (using LDpred) for BMI, and from 2.27% to 3.37% (using 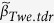) and 3.62% (using LDpred) for DBP. 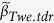 and LDpred were the best performing methods overall if we consider the maximum *R*^2^ achieved across different *p* thresholds. The performances of these two methods were largely comparable over most traits. LDpred outperformed 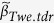 for a larger margin on weight while it performed considerably worse than other methods on the prediction of INS.

Another major focus of this study is the performance of different PRS weighting schemes when *all* markers were included. Similar to our simulation results, 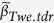 unanimously outperformed other estimators in this case. In addition, for 11 out of the 12 traits studied (with the exception of INS), the prediction *R*^2^ from 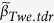 using all markers outperformed the best *R*^2^ from standard PRS.

### Computational speed of different methods

For the different methods mentioned above, we recorded the time taken to compute the adjusted regression weights 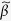 over 20 simulations (5 × 4 CV) for “scenario 1” (listed in Supplementary Table 3). It is clear that LDpred is the most computationally intensive owing to the need for MCMC procedures (Table 5). The other methods took less than 45 seconds to compute the regression weights for 20 simulations while LDpred took ~4.7 days. It is worth noting that the number of genetic variants is modest in this study (334458 variants), and we expect the runtime of LDpred to increase further with larger panels of markers.

## Discussion

In this study we proposed empirical Bayes approaches to estimating the underlying effect sizes of genetic variants, and made use of these estimates for new ways of constructing PRS. As for the predictive ability of the newly proposed PRS, the empirical Bayes versions of PRS in general lead to improvement in performance, both if we consider the PRS from the best *p*-value threshold or when all markers are included. Remarkably, for almost all traits studied in the NFBC, the predictive performances using 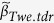 with *all* SNPs outperformed the standard PRS at the optimal *p* thresholds. We also observed that the newly proposed estimator 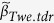 had largely comparable performances with LDpred for most traits, but the computational speed is much faster and no *p* thresholds need to be chosen.

In the simulations with independent SNPs, we employed a simple yet commonly used form of distribution for the regression coefficient, assuming a normal distribution of mean zero and variance equal to mean heritability explained. In reality this assumption might not hold as the actual distribution of the effect sizes can take any form and would likely vary with different traits. While we did not see much improvement in the maximum prediction *R*^2^ in these simulations, the results showed that the empirical Bayes methods performed well when *all* markers were included, equivalent to using a single *p* threshold of one.

We then simulated a wider range of genetic architecture with real genotype data. As expected, LDpred performed well under an infinitesimal model with a matching Gaussian prior. The empirical Bayes methods may over-correct the effect sizes under a non-sparse scenario, leading to worse performances. Under another model where there were only a few large effects, all correction methods outperformed standard PRS and the results were quite close. Nevertheless these two kinds of genetic architecture might be less likely than a mixture of small and larger effects. Under this third model, we observed mixed results of different estimators. LDpred tends to perform worse at lower levels of heritability (e.g. *h*^2^ = 0.1) but generally better at higher *h*^2^. A likely explanation is that the effect sizes may be too small in relation to the sampling error of the PRS at low heritability levels. As LDpred includes all markers in prediction, the sampling error may be larger than the pruned PRS derived from fewer markers.

A recent study by Dudbridge *et al*.^19^ estimated that the differences in prediction performances between pruned scores and jointly modelled scores were likely to be small at sample sizes up to 100,000. The small difference is likely due to the extra sampling error incurred by joint modeling of large number of markers. Dudbridge *et al*.^19^ also noted that the largest improvements by joint modelling (such as LDpred) tend to be in autoimmune diseases (*e.g*. multiple sclerosis, rheumatoid arthritis, type I diabetes) which are associated with HLA variants. Multiple large-effect variants in LD are likely to be present within the HLA region for these diseases. For other traits, there may not be multiple strong effects within a LD region; hence the improvement by joint modeling may be less marked.

The applications of the PRS methodologies to actual cardio-metabolic traits showed superior performances of 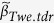 and LDpred. Although LDpred performed both shrinkage and LD modelling, the performances of 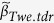 were comparable for most traits. The tradeoff between inclusion of more markers and sampling errors, as pointed out by Dudbridge *et al*.^19^, may partially account for this result. Another possible reason is that LDpred may perform less well for some traits with genetic architecture that differs from a point-normal prior.

It is also noteworthy that for methods that rely on an external LD reference panel, there is a certain level of approximation which may affect the predictive performance. In this study, the LD reference data was derived from the original sample by CV, hence LD mismatch should not be a problem; however it may not be true for other studies using other reference datasets. LD pruning or clumping methods may be advantageous when there is insufficient sample size to derive a reference LD matrix, because inaccurate LD information may degrade the performance of LD-dependent methods. For example, authors of LDpred suggest the reference panel should contain at least 1000 unrelated individuals with the same ancestry make-up as the people from which the summary statistics are derived. This may not always be feasible depending on the population under study. In addition, it may be difficult to find genotype data that matches very well with the LD pattern of the original study population (from which summary statistics are derived). In spite of the above arguments, joint modelling methods such as LDpred may become more useful when sample sizes of studies continue to rise. Our proposed methodology does not fully account for LD, and may be improved by modelling LD structure for the whole panel of markers.

A remarkable feature we wish to highlight is that the predictive performance of the empirical Bayes method 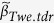 maintains despite escalating *p*-value thresholds, and essentially a single threshold of one can be chosen. In other words, the new estimator 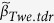 enables an automatic PRS weighting scheme without the need of choosing tuning parameters (such as *p*-value thresholds or fraction of causal variants). This is a unique feature among all PRS weighting methods.

What are the advantages for having a method that does *not* require the choice of tuning parameters? Firstly, it avoids a potential bias in the estimation of the true prediction errors. Varma and Simon^20^ showed that if one performs CV to choose the optimal tuning parameters and reports the resulting best prediction error from the same CV, this error estimate is subject to bias on the optimistic side. They advised a nested CV procedure, where an inner loop is used to tune the algorithm parameters, and an outer loop is used to estimate the prediction error. In this case the dataset used for computing prediction error is not used for any parameter tuning. As the main aim in this paper is to compare different estimators (which are subject to the same kind of bias) and nested CV is computationally intensive, we simply report the best predictive performances across different *p*; the same approach was taken in the study on LDpred^9^ and in Mak *et al*.^10^. Nevertheless, if the aim of a study is to obtain a precise and unbiased estimate of predictive ability (*e.g.* to assess if a PRS can be used in clinical practice to predict future disease risks), then this potential bias should preferably be corrected by a nested CV or estimating the prediction error in an independent sample. The former however is not possible if only summary statistics are available while the latter may not be practical if the study involves an uncommon disease phenotype or treatment side effect for example. Designating a portion of the testing sample for tuning will impair the precision of prediction error estimates. Clearly an automatic PRS weighting scheme avoids the above complications. Essentially such a weighting scheme saves a portion of the sample size that is required for parameter tuning.

The second advantage is that computational load can be reduced when no parameters need to be tuned. Also, the choice of *p* thresholds is usually arbitrary. A diligent search across many thresholds will increase computational cost while the optimal threshold may be missed if too few thresholds are tried. When only summary statistics are available, the automatic weighting scheme is also directly applicable to a single new patient. This is not true for the other methods in general as parameter tuning requires a certain sample size. While one can choose a pre-determined threshold, using an non-optimized threshold might lead to inferior predictive performance.

Another important feature of the proposed methodology is that it is computationally simple and conceptually relatively straightforward. Compared to LDpred, it does not require MCMC procedures. As only summary statistics are required, it can be readily applied to a wide variety of traits and take advantage of the large sample sizes in GWAS meta-analyses. The empirical Bayes approach also does not require any particular priors to be specified or assumptions about the genetic architecture of the trait, such as the proportion of causal markers or distribution of effect sizes. On the other hand, LDpred requires the assumption of a point-normal mixture prior.

The key limitation of our proposed methodology, as already discussed earlier, is that we do not fully account for the LD structure of all markers. It is also worth mentioning that if raw genotype data is available, other methods such as those based on a mixed effects models^21^ or regularized regression approaches^22^ may be superior, although the computational cost may be high if all genotypes are fit. In this study we have assumed that population stratification has been adequately controlled for, otherwise the predictive performance may be impaired. Another limitation is that the newly proposed PRS scoring method may not perform uniformly well under all genetic architectures. In simulations and the NFBC study, we observe that LDpred performed better in some scenarios, although the differences were in general not large. For the majority of the cardio-metabolic traits under study, the newly proposed PRS outperformed the standard PRS approach at the best *p*-value threshold. Nevertheless, for fasting insulin, which has a very low prediction *R*^2^, the performance of 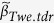 was less satisfactory than that for other traits. It remains a significant challenge to derive a methodology that performs well for all types of genetic architecture. It will also be desirable to test the proposed methodologies on a wider range of complex traits and on summary statistics from larger samples.

In summary, we have developed a new empirical Bayes framework to improve risk prediction from polygenic scores, using summary statistics. We hope the presented methodology will be a useful new way to improve genomic risk prediction, with the potential for translation to better personalized medical care in the future.

## Additional Information

**How to cite this article:** So, H.-C. and Sham, P. C. Improving polygenic risk prediction from summary statistics by an empirical Bayes approach. *Sci. Rep.* **7**, 41262; doi: 10.1038/srep41262 (2017).

**Publisher's note:** Springer Nature remains neutral with regard to jurisdictional claims in published maps and institutional affiliations.

## Supplementary Material

Supplementary Information

## Figures and Tables

**Table 1:** The predictive performances (in *R*^2^ for linear traits and AUC for binary traits) at the optimal p-value threshold for standard PRS and three other weighting schemes in simulations for *N* = 5000 and 10000 using independent SNPs.

*N*	*h*^*2*^	Linear traits				Binary traits			
		Standard	Tdr	Tweedie	Tweedie*tdr	Standard	Tdr	Tweedie	Tweedie*Tdr
5000	0.15	0.048	0.054	0.052	0.047	0.546	0.544	0.544	0.536
	0.35	0.204	0.216	0.205	0.200	0.608	0.621	0.616	0.605
	0.55	0.399	0.410	0.398	0.397	0.691	0.705	0.702	0.690
10000	0.15	0.081	0.086	0.084	0.081	0.57	0.576	0.574	0.567
	0.35	0.274	0.279	0.267	0.266	0.679	0.688	0.687	0.682
	0.55	0.468	0.473	0.457	0.457	0.763	0.771	0.771	0.768

We first applied LD-clumping with an r^2^ threshold of 0.25 to all SNPs, followed by p-value thresholding in the testing set. The results were derived from testing over a range of p-value thresholds and picking the threshold that gave the best predictive performance.

*N* denotes the total sample size. For binary traits, an equal number of cases and controls are simulated (e.g. for *N* = 5000, there are 2500 cases and 2500 controls). Tdr: True discovery rate; *h*^*2*^: total heritability explained. The rest of the simulation results are presented in Supplementary Table 1.

**Table 2:** The predictive performances (in *R* ^2^ for linear traits and AUC for binary traits) when all markers are included in PRS in simulations for *N* = 5000 and 10000 using independent SNPs.

*N*	*h*^*2*^	Linear traits					Binary traits				
		Standard	Tdr	Tweedie	Tweedie*tdr	Standard best *p*	Standard	Tdr	Tweedie	Tweedie*tdr	Standard best *p*
5000	0.15	0.012	0.053	0.040	0.047	0.048	0.527	0.542	0.530	0.535	0.546
	0.35	0.051	0.214	0.187	0.200	0.204	0.564	0.620	0.595	0.605	0.608
	0.55	0.118	0.407	0.377	0.397	0.399	0.599	0.705	0.680	0.690	0.691
10000	0.15	0.019	0.085	0.074	0.081	0.081	0.537	0.576	0.559	0.567	0.570
	0.35	0.087	0.275	0.255	0.266	0.274	0.585	0.688	0.671	0.682	0.679
	0.55	0.187	0.461	0.444	0.457	0.468	0.632	0.770	0.757	0.768	0.763

For the columns labelled “Standard”, “Tdr”, “Tweedie” and “Tweedie*tdr”, we first applied LD-clumping with an *r*^2^ threshold of 0.25 to all SNPs, then PRS was derived using *all* SNPs that remained. There was *no* selection of *p*-value thresholds.

The best predictive performance obtained from optimal *p*-value thresholds using standard PRS are also shown for comparison (under the column “standard best *p”*). *N* denotes the total sample size. For binary traits, an equal number of cases and controls are simulated. Tdr: True discovery rate; *h*^*2*^: total heritability explained. The rest of the simulation results are presented in Supplementary Table 2.

**Table 3:** Predictive performances (prediction *R* ^2^ in %) of the standard PRS and four other PRS schemes in simulations using real genotype data (a mixture small and large effects simulated).

% casual	*h*^2^	Type	Standard	Tdr	Tweedie	Tweedie*tdr	LDpred
0.1%	10%	max	0.700	**0.962**	0.951	0.925	0.705
		all SNPs	0.044	0.644	0.165	**0.903**	0.055
0.1%	20%	max	2.497	2.712	2.669	2.922	**3.192**
		all SNPs	0.072	2.025	0.178	**2.909**	0.041
0.1%	30%	max	6.622	6.800	6.627	7.079	**7.376**
		all SNPs	0.411	4.342	2.852	**7.079**	0.489
0.1%	40%	max	7.327	8.289	8.522	**8.846**	7.743
		all SNPs	0.265	5.048	2.634	**8.790**	0.817
0.25%	10%	max	0.921	1.154	1.269	**1.328**	0.986
		all SNPs	0.075	0.864	0.237	**1.327**	0.162
0.25%	20%	max	2.381	**2.446**	2.342	2.432	0.913
		all SNPs	0.038	2.099	0.320	**2.416**	0.038
0.25%	30%	max	2.015	2.469	2.328	2.571	**2.805**
		all SNPs	0.730	1.899	1.268	**2.571**	0.653
0.25%	40%	max	4.762	5.328	5.218	5.333	**5.737**
		all SNPs	0.910	3.707	2.882	**5.333**	1.136
1.0%	10%	max	0.695	**0.800**	0.773	0.766	0.374
		all SNPs	0.069	0.498	0.102	**0.740**	0.056
1.0%	20%	max	1.439	**1.513**	1.350	1.206	1.253
		all SNPs	0.042	0.915	0.132	**1.153**	0.040
1.0%	30%	max	3.018	3.189	3.274	**3.369**	2.656
		all SNPs	0.274	3.189	1.059	**3.367**	0.730
1.0%	40%	max	2.906	3.104	3.011	**3.407**	3.404
		all SNPs	0.599	2.147	1.565	**3.407**	1.178
2.5%	10%	max	0.730	0.934	0.982	**1.007**	0.960
		all SNPs	0.035	0.387	0.112	**0.977**	0.046
2.5%	20%	max	1.912	2.106	2.096	2.045	**2.132**
		all SNPs	0.352	1.452	0.797	**2.045**	0.338
2.5%	30%	max	1.269	1.398	1.440	1.482	**1.712**
		all SNPs	0.353	0.972	0.648	**1.482**	0.324
2.5%	40%	max	2.691	2.861	2.787	2.925	**3.651**
		all SNPs	0.706	2.043	1.554	**2.924**	0.605

A mixture of small and large effects was simulated as described in the main text. The best performing PRS weighting method in each scenario is in bold. % causal: percentage of causal markers; *h*^2^: total heritability explained by panel markers. For all methods except LDpred, we first applied LD-clumping with an *r*^2^ threshold of 0.25 to all SNPs. “Max” refers to the maximum prediction *R*^2^ achieved after optimizing over a range of *p*-value thresholds or fractions of causal variants. “All.SNPs” refers to the predictive performance using all SNPs after LD-clumping, except for LDpred where no clumping was performed. All predictive performances were measured by *R*^2^ in %.

**Table 4:** Predictive performances (prediction *R* ^2^ in %) of the standard PRS and four other PRS schemes, applied to twelve cardio-metabolic traits in the Northern Finland Birth Cohort.

		Standard	Tdr	Tweedie	Tweedie*tdr	LDpred
LDL	max	3.731	3.956	4.349	**4.722**	4.070
	all SNPs	0.166	1.873	1.295	**4.711**	1.295
HDL	max	10.050	10.436	10.325	**10.463**	10.438
	all SNPs	0.311	6.788	3.548	**10.247**	0.472
TG	max	2.256	2.627	2.784	**2.835**	2.508
	all SNPs	0.056	1.128	0.311	**2.707**	0.130
FG	max	1.234	1.239	1.775	**1.824**	1.708
	all SNPs	0.180	0.800	0.519	**1.792**	0.162
BMI	max	0.481	0.682	0.984	1.116	**1.166**
	all SNPs	0.207	0.640	0.442	**1.116**	0.339
WHR	max	18.764	19.449	20.051	20.231	**21.236**
	all SNPs	0.044	18.916	7.139	**20.231**	0.019
INS	max	0.167	0.183	0.136	**0.153**	0.048
	all SNPs	0.044	0.117	0.028	**0.127**	0.026
CRP	max	0.644	0.700	**0.894**	0.787	0.740
	all SNPs	0.166	0.140	0.318	**0.731**	0.102
SBP	max	7.641	8.124	8.519	8.545	**9.705**
	all SNPs	0.132	4.853	2.565	**8.519**	0.291
DBP	max	2.270	2.842	3.229	3.369	**3.620**
	all SNPs	0.359	1.684	1.636	**3.369**	0.340
Height	max	29.907	30.258	31.065	**31.167**	NA^
	all SNPs	0.168	20.153	12.318	**31.167**	NA^
Weight	max	12.708	13.354	13.973	14.093	**18.225**
	all SNPs	0.106	12.244	6.881	**14.093**	0.049

The best performing PRS weighting method in each scenario is in bold. Max: maximum prediction *R*^2^ achieved after optimizing over a range of *p*-value thresholds or fractions of causal variants; all SNPs: predictive performance using all SNPs. Note that for all methods except LDpred, the original set of genome-wide SNPs were LD-clumped by PLINK before construction of PRS.

LDL: Low density lipoprotein; HDL, high density lipoprotein; TG, triglyceride; FG, fasting glucose; BMI, body mass index; WHR, waist-hip ratio; INS, fasting insulin; CRP, C-reactive protein; SBP, systolic blood pressure; DBP, diastolic blood pressure.

^The LDpred program failed to run properly for the prediction of height despite repeated trials; hence it is listed as NA in this table.

**Table 5:** Computational speed of different algorithms.

Method	Average time taken for 1 simulation	Time taken for 20 simulations
Tdr	0.14 s	2.73 s
Tweedie	1.34 s	26.74 s
Tweedie *tdr	2.05 s	40.98 s
LDpred	5 h 37 min 44 s	112 h 34 min 32 s

We compared the time taken for computing the corrected effect sizes based on one simulation scenario. Names of methods are defined as in previous tables; h: hours; min: minutes; s, seconds.
