# Supplementary material for: Improving polygenic risk prediction from summary statistics by an empirical Bayes approach
Source: Sci Rep. 2017 Feb 1;7:41262. doi: 10.1038/srep41262 (PMC5286518; doi:10.1038/srep41262)
Supplement: Supplementary Information [file srep41262-s1.pdf]

Supplementary Information

Improving polygenic risk prediction from summary statistics by an empirical Bayes approach

Hon-Cheong So and Pak C. Sham

Supplementary Table 1 Predictive performances (in R^2 for linear traits and AUC for binary traits) *at the optimal p-value threshold* for standard PRS and three other weighting schemes in simulations, for N = 15000 and 20000

		Linear traits				Binary traits			
N	h^2	Standard	Tdr	Tweedie	Tweedie*tdr	Standard	Tdr	Tweedie	Tweedie*Tdr
15000	0.15	0.104	0.108	0.104	0.102	0.591	0.599	0.598	0.591
	0.35	0.303	0.306	0.294	0.293	0.708	0.714	0.714	0.710
	0.55	0.500	0.504	0.491	0.490	0.793	0.799	0.799	0.797
20000	0.15	0.112	0.114	0.110	0.110	0.607	0.613	0.612	0.609
	0.35	0.312	0.312	0.304	0.302	0.723	0.728	0.728	0.726
	0.55	0.514	0.516	0.500	0.498	0.811	0.814	0.814	0.814

We first applied LD-clumping with an r^2 threshold of 0.25 to all SNPs, followed by p-value thresholding in the testing set. The results were derived from testing over a range of p-value thresholds and picking the threshold that gave the best predictive performance.

N denotes the total sample size. For binary traits, an equal number of cases and controls are simulated. Tdr: True discovery rate; h^2: total heritability explained.

Supplementary Table 2 Predictive performances (in R^2 for linear traits and AUC for binary traits) when *all* markers are included in PRS in simulations for N = 15000 and 20000

		Linear traits					Binary traits				
N	h^2	Standard	Tdr	Tweedie	Tweedie*tdr	Standard best p	Standard	Tdr	Tweedie	Tweedie*tdr	Standard best p
15000	0.150	0.025	0.106	0.096	0.102	0.104	0.545	0.599	0.586	0.591	0.591
	0.350	0.112	0.301	0.284	0.293	0.303	0.601	0.714	0.703	0.710	0.708
	0.550	0.235	0.487	0.483	0.490	0.500	0.656	0.799	0.792	0.797	0.793
20000	0.150	0.032	0.114	0.104	0.110	0.112	0.552	0.613	0.602	0.609	0.607
	0.350	0.133	0.306	0.298	0.302	0.312	0.616	0.727	0.720	0.726	0.723
	0.550	0.274	0.497	0.494	0.498	0.514	0.673	0.813	0.806	0.814	0.811

For the columns labelled “Standard”, “Tdr”, “Tweedie” and “Tweedie*tdr”, we first applied LD-clumping with an r^2 threshold of 0.25 to all SNPs, then PRS was derived using *all* SNPs that remained. There was *no* selection of p-value thresholds.

The best predictive performance obtained from optimal p-value thresholds using standard PRS are also shown for comparison (under the column “standard best p”). N denotes the total sample size. For binary traits, an equal number of cases and controls are simulated. Tdr: True discovery rate; h^2: total heritability explained.

Supplementary Table 3 Details of simulation scenarios with a mixture of small and larger effects

Scenario	Fraction of causal variants (%)	Total heritability explained (%)	No. of variants simulated under Vg ~ uniform(0.4%, 0.8%)
1	0.10	10	5
2	0.10	20	5
3	0.10	30	5
4	0.10	40	5
5	0.25	10	10
6	0.25	20	10
7	0.25	30	10
8	0.25	40	10
9	1.00	10	15
10	1.00	20	15
11	1.00	30	15
12	1.00	40	15
13	2.50	10	20
14	2.50	20	20
15	2.50	30	20
16	2.50	40	20

Please refer to the main text for details. We simulated two sets of casual variants and then combined them. The first set of casual variants were simulated from a double exponential distribution and then scaled. For the second set of variants, their variance explained (Vg) was assumed to follow a uniform distribution in the interval [0.4%, 0.8%].

Supplementary Table 4 Predictive performances (prediction R^2 in %) of the standard PRS and four other PRS schemes in simulations using real genotype data under an infinitesimal model

h^2	Type	Standard	Tdr	Tweedie	Tweedie*tdr	LDpred
10%	max	0.123	0.090	**0.190**	0.154	0.172
	all SNPs	0.045	0.071	0.050	**0.099**	0.087
20%	max	0.243	0.092	0.130	0.101	**0.407**
	all SNPs	0.239	0.030	0.109	0.078	**0.407**
30%	max	0.687	0.287	0.435	0.095	**0.935**
	all SNPs	0.686	0.287	0.410	0.078	**0.923**
40%	max	1.230	0.750	0.931	0.297	**1.794**
	all SNPs	1.223	0.750	0.931	0.297	**1.683**

We simulated an infinitesimal model in which all markers were causal and their effects followed a normal distribution of zero mean. The best performing PRS weighting method in each scenario is in bold. % causal: percentage of causal markers; h^2: total heritability explained by panel markers. For all methods except LDpred, we first applied LD-clumping with an r^2 threshold of 0.25 to all SNPs.

"Max" refers to the maximum prediction R^2 achieved after optimizing over a range of *p*-value thresholds or fractions of causal variants. "All.SNPs" refers to the predictive performance using all SNPs after LD-clumping, except for LDpred where no clumping was performed. All predictive performances were measured by R^2 in %.

Supplementary Table 5 Predictive performances (prediction R^2 in %) of the standard PRS and four other PRS schemes in simulations using real genotype data under a model of large-effect variants only

No. of large-effect variants	Total h^2 explained	Type	Standard	Tdr	Tweedie	Tweedie*tdr	LDpred
5	3%	max	1.913	2.286	2.262	2.276	**2.406**
		all SNPs	0.120	1.796	0.645	**2.265**	0.100
10	6%	max	2.830	**3.024**	2.938	2.941	2.295
		all SNPs	0.065	1.946	0.393	**2.896**	0.023
15	9%	max	4.976	5.209	4.658	4.850	**5.292**
		all SNPs	0.172	3.418	1.581	**4.830**	0.115
20	12%	max	8.456	8.655	8.430	8.452	**9.306**
		all SNPs	0.053	3.828	1.567	**8.119**	0.254

We simulated a model in which there was a limited number of large-effect variants each with heritability explained of 0.6%. All other markers were null. The best performing PRS weighting method in each scenario is in bold. % causal: percentage of causal markers; h^2: total heritability explained by panel markers. For all methods except LDpred, we first applied LD-clumping with an r^2 threshold of 0.25 to all SNPs.

"Max" refers to the maximum prediction R^2 achieved after optimizing over a range of p-value thresholds or fractions of causal variants. "All.SNPs" refers to the predictive performance using all SNPs after LD-clumping, except for LDpred where no clumping was performed. All predictive performances were measured by R^2 in %.
